# Lachesis and Lycopodium

**Published:** 1893-05

**Authors:** 


### HOMŒOPATHIC PHYSICIAN,

A MONTHLY JOURNAL OF

HOMŒOPATHIC MATERIA MEDICA AND CLINICAL MEDICINE.

"If our school ever gives up the strict inductive method of Hahnemann, we are lost, and deserve only to be mentioned as a caricature in the history of medicine."—CONSTANTINE HERING.

Vol. XIII. MAY, 1893. No. 5.

### EDITORIAL.

LACHESIS AND LYCOPODIUM.—We publish this month an instructive comparison of the throat symptoms of Lachesis and Lycopodium, by Dr. E. V. Ross, and copied from *The Homœopathic World.* No two remedies in the materia medica have excited more opposition to their introduction and more ridicule and contempt. Yet they are simply invaluable, and it would be difficult for us, as consistent homœopathists, to succeed without their agency.

The idea of supplying the whole world with medicine from a single drop of the poison of the Lachesis snake seemed particularly absurd to a large portion of the homœopathic profession, especially those who dealt only in crude doses, and they obstinately refused to prescribe the new drug. Meanwhile, those of the school who really understood the full significance of the homœopathic principle, and had access to the monograph containing the pathogenesis of Lachesis, one of the noblest memorials the late Dr. Hering has left of himself, were favored with a perfect revelation of its virtues in the cure of all sorts of sick conditions, but especially of sore throat.

Lycopodium, also, was opposed, because it seemed to be impossible to derive symptoms from an inert powder. Yet, as was well said by Dr. J. B. Bell, "Lycopodium is one of the noblest monuments to the genius of Hahnemann, as well as one of the most convincing proofs of the truth of the homœopathic doctrines."

As shown in the article to which we have referred, Lachesis and Lycopodium are complementary to each other. This is true not alone in throat diseases, but in many other maladies. Take one distinguished symptom as an illustration. In Lachesis the symptoms go from left to right. In Lycopodium they go in the opposite direction. This peculiarity of direction is one of the most certain indications for these remedies respectively. Many other remedies have some left-to-right and right-to-left conditions. A list of these was published in this journal for March, 1891, page 107. But the value of other drugs for these indications does not compare with Lachesis and Lycopodium, which are simply marvelous in their effects. I have seen the most wonderful cases of erysipelas of the face going from right to left, with large abscesses formed, and brain symptoms beginning, quickly relieved by a few doses of Lycopodium. This experience I have had, not once, but many times. Tonsillitis beginning on the right side and going to the left I have repeatedly relieved with Lycopodium, and either caused it to abort, or, if it had gone too far for that, hastened the bursting of the abscess and the return to complete health. The same is true of Lachesis. Take its action in cases of violent dyspnœa, for instance. A man in the last stages of consumption, throwing himself wildly about the bed in a fierce and desperate struggle for breath, has been instantly relieved by Lachesis, and kept quiet for two weeks afterward, enabling him to write his will, and finally to pass away peacefully and unconsciously. Every physician practicing the Hahnemannian method has had similar experiences. The characteristic aggravation after sleep should be remembered. As Dr. Guernsey used to express it, "the patient sleeps into the aggravation." This was his keynote for Lachesis. It is, therefore, important that these two remedies should be most carefully studied, and their most valuable symptoms kept vividly in mind.

